# Ano‐rectovaginal fistula after obstetrical anal sphincter injury: Diverting stoma does not improve the surgical results

**DOI:** 10.1111/codi.16211

**Published:** 2022-06-21

**Authors:** Aurélien Venara, Bertrand Trilling, Marie Ngoma, Charlène Brochard, Emilie Duchalais, Laurent Siproudhis, Jean‐Luc Faucheron, Vincent de Parades, Arnaud Alves, Eddy Cotte, Mehdi Ouaissi, Valérie Bridoux, Lisa Corbière, Jeanne Heraud, Pablo Ortega‐Deballon, Fawaz Abo‐Alhassan, Jean‐Francois Hamel

**Affiliations:** ^1^ Department of Digestive Surgery University Hospital of Angers Angers Cedex 9 France; ^2^ CHU Angers University of Angers Angers France; ^3^ CHU Angers HIFIH, SFR ICAT University of Angers Angers France; ^4^ TIMC‐IMAG Laboratory, National Centre for Scientific Research, Grenoble National Polytechnical Institute, Grenoble Alps University Hospital Grenoble Alps University Grenoble France; ^5^ Colorectal Surgery Unit, Visceral surgery and Acute Care surgery Department Grenoble Alps University Hospital Grenoble France; ^6^ Institut Léopold Bellan, Groupe Hospitalier Paris Saint‐Joseph Service de Proctologie Médico‐Chirurgicale Paris France; ^7^ Unité D'explorations Fonctionnelles Digestives CHU Rennes Pontchaillou Rennes France; ^8^ Unité de Proctologie, Service des Maladies de l'appareil digestif CHU Rennes Pontchaillou Rennes France; ^9^ Department of Digestive Surgery University Hospital of Nantes Nantes France; ^10^ Department of Digestive Surgery University Hospital of Caen Caen Cedex France; ^11^ UMR INSERM U1086 Anticipe Centre François Baclesse Caen Cedex France; ^12^ Department of Digestive Surgery, Hôpital Lyon Sud CHU Lyon Cedex France; ^13^ Faculty of Medicine of Lyon Sud‐Charles Mérieux University Lyon 1 Cedex France; ^14^ Department of Digestive, Oncological, Endocrine, Hepato‐Biliary Pancreatic and Liver Transplant Surgery Trousseau Hospital Chambray les Tours France; ^15^ Department of Digestive Surgery Rouen University Hospital Rouen France; ^16^ Department of Digestive Surgery CHU Rennes Pontchaillou Rennes France; ^17^ Department of Gynecology and Obstetrics University Hospital of Angers Angers Cedex 9 France; ^18^ Department of Digestive Surgery Dijon University Hospital Dijon France; ^19^ Department of Biostatistics. La Maison de la Recherche University Hospital of Angers Angers Cedex 9 France

**Keywords:** ano‐rectovaginal fistula, diverting stoma, management, obstetrical anal sphincter injury

## Abstract

**Aim:**

Ano‐rectovaginal fistulas (ARVF) are challenging for the surgeon. Most of the series mix aetiologies, leading to confusion with respect to the conclusion. The aim of this study was to assess the factors associated with the success of ARVF management following obstetrical anal sphincter injury (OASIS).

**Methods:**

This retrospective multicentric study included all the patients undergoing surgery for ARVF identified by the hospital codes. Patients for whom the aetiology of ARVF was not OASIS were excluded. The major outcome measure was the success of the procedure.

**Results:**

Sixty patients with treated ARVF due to OASIS were identified. The success of overall management was 91.7%. Female patients underwent a mean of 2.5 (±1.7) procedures. A diverting stoma was formed in 29 patients (48.3%) of which 26 were closed at the end of the management period (89.7%). Of the 148 surgical procedures, only 55 were successful (37.2%). The order of the procedures (OR = 1.38; 95% CI: 0.75–2.51) or the diverting stoma (OR = 1.46; 95% CI: 0.31–6.91) were not significantly associated with the success of the surgery. However, Martius flap (OR = 4.13; 95% CI: 1.1–15.54) and Musset procedures (OR = 5.79; 95% CI: 1.77–18.87) produced better results than the endorectal advancement flap (ERAF). The other procedures did not show a significant correlation with management success.

**Conclusion:**

A diverting stoma is not mandatory in the management of ARVF due to OASIS to improve the success of the surgical procedure. While the Martius flap procedure offers better results, the ERAF procedure may be preferred as a primary intervention in the absence of sphincter injury as it is less invasive. In cases of residual sphincter injury, the Musset procedure is most likely to be the best option.

**What does this study contribute to the literature?**

Obstetrical ano‐rectovaginal fistula is challenging for the surgeon and diverting stoma is often discussed as a means of improving surgical results. Our result advocates for the inefficiency of diverting stoma to improve the success of the surgical procedure.

## INTRODUCTION

Obstetrical anal sphincter injury (OASIS) occurs in 0.25% to 6% of vaginal deliveries around the world [1]. Even if OASIS is reported to have a good prognosis, it can have some short‐, mid‐ and long‐term consequences [2]. OASIS leads not only to an increased risk of anal incontinence by 2.27 to 3.97‐fold after 20 years [3], but also to the risk of ano‐rectovaginal fistula (ARVF). ARVF incidence is reported in 0.2 to 4 per 1000 deliveries [4] and in 1%–3% following OASIS [5].

This latter complication associated with OASIS is challenging for the colorectal surgeon to manage because of the high failure rate of surgical procedures [6]. Indeed, Fu et al. [7] report an overall success rate of 71.2% per fistula closure procedure, but only 55.5% after the first procedure. Other authors state that the initial success rate of ARVF management is relatively low, having been reported as 60% [8], and the best sequence of surgical repair is unknown.

Mucosal flap or primary closure and sphincteroplasty are associated with a high success rate [4, 9] and the American Society of Colon and Rectal Surgeons (ASCRS) recommends using endorectal advancement flap with or without sphincteroplasty as a primary intervention. However, this recommendation is based on low‐quality evidence [10].

As the success of management is determined by numerous variables including the aetiology [11], the literature is difficult to analyse because studies comparing the different repair techniques and the need for diverting stoma generally include cases associated with many different aetiologies [6, 8, 9, 12, 13], while the prognosis for obstetric and nonobstetric ARVF differs [14].

Specific assessment of ARVF management following OASIS is necessary in order to provide strong evidence on which to base recommendations regarding the best procedure or the need for a diverting stoma.

The aim of this study was to assess the factors associated with the success of ARVF management following OASIS.

## MATERIALS AND METHODS

A retrospective cohort study including all patients referred for ano‐rectovaginal fistula was conducted in 10 centres participating in the study. Adult patients were included if they underwent surgery for anorectovaginal fistula, anorectovulvar fistula, or fistula between the lower rectum and the vagina between 2005 and 2020. The surgical procedure was performed by colorectal surgeons or by proctologists. Patients were included only if the fistula was due to obstetrical anal sphincter injuries (OASIS).

Patients were excluded if the fistula had a cause other than OASIS, if no surgical procedure was performed to close the fistula or if the patient expressed her opposition to participating in retrospective studies during her hospitalization.

The study received the approval of our local ethical committee (2021/034) and the database was declared to the Commission Nationale Informatique & Libertés (France's national data protection agency; ar21‐0014v0).

Patients were selected according to reason for hospital admission. The codes for anal fistula were used (N82.1 / N82.3/ N82.4/ K60.4/ N82.9/ O23.5/ N99.8 ± K603). The following data were collected in an anonymized database:

1. Demographic data: age, body mass index (BMI), medical history, number of pregnancies
2. Aetiology of the ARVF
3. Type of surgery performed, order of the surgeries
4. Creation of a diverting stoma
5. Efficacy of the surgery
6. Follow‐up after the last surgery

The main outcome measure was the success of the surgery. Success of the surgery was confirmed by a clinical examination and defined by the absence of ano‐rectovaginal suppuration and of stools or gas in the vagina. The successful intervention was the last reparatory surgery in the management period. Patients could undergo a final surgery after the successful intervention to close the stoma.

If the patient underwent a definitive stoma procedure to treat the suppuration, the management was not considered successful.

### Management of the patients

All the centres involved were university hospitals with colorectal surgeons or proctologist surgeons and most reported at least 30 ARFV procedures during the study period. Patients could be primarily or secondarily referred to the centre.

A diverting stoma could be performed at any time during patient management, according to the surgeon's practice and the symptoms of the patients. The timing of the diverting stoma performance was collected (rank of the surgery). The order in which the procedures were performed and the choice of the procedure were also left to the surgeon's discretion, according to the size of the defect and to the presence of a sphincter tear. The first procedure was usually sphincter‐sparing as recommended by the ASCRS guidelines [10]. More aggressive procedures were chosen as a secondary phase.

There was no standard protocol between the centres concerning perioperative management. Prophylactic antibiotics could be used as a single perioperative injection or during three postoperative days, according to the surgeon's practice, in order to limit the risk of abscess formation. Vaginal oestrogen was not recommended and laxatives were recommended for at least 15 postoperative days.

### Statistical analysis

Continuous covariates were described using mean and standard deviations, and compared using Mann & Whitney tests, whereas categorical covariates were described using percentages, and compared using Fisher's exact tests.

As the observations could be repeated for the same patients due to the recurrences of the ano‐rectovaginal fistula, the factors associated with a change in surgery effectiveness were studied through a mixed logistic model regarding patients as a random effect covariate. In the estimation process, the variance–covariance structure was defined as being unstructured. The different fixed‐effect covariates taken into consideration in the model were the BMI of the patients, the number of recurrences prior to the considered surgical treatment, the type of surgery performed, the joint formation of a diverting stoma and the interaction between the number of recurrences prior to the considered surgical treatment and the diverting stoma formation. The study of the impact of such a diverting stoma could differ depending on the number of recurrences. No interaction was observed between the type of surgery and the number of recurrences due to the large number of surgical procedures and the relatively small number of patients. The impact of a given surgical technique was then assumed to be constant depending on the number of recurrences.

All the statistical analyses were performed assuming a type I error probability of 0.05.

## RESULTS

**FIGURE 1:**
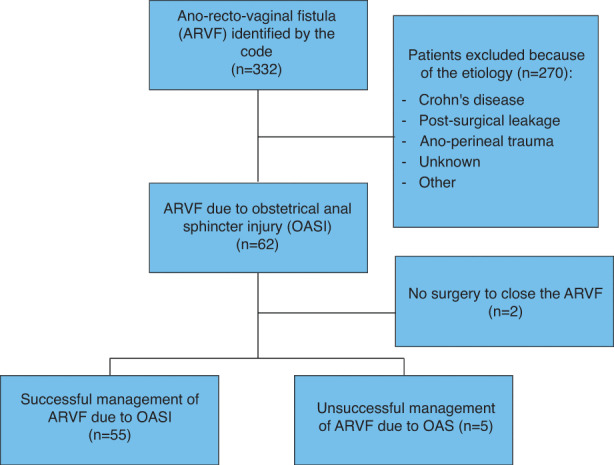
Flow chart of our population

A total of 332 patients with ARVF were identified in this cohort. Several aetiologies were found, such as OASIS (*n* = 62; 18.7%), Crohn's disease (*n* = 76; 22.9%), post‐surgical leakage (*n* = 105; 31.6%), anoperineal trauma (*n* = 5; 1.5%) or other (Figure 1). Other included cancer (*n* = 41), unknown or cryptogenic (*n* = 31), treatment with Nicorandil (*n* = 1), diverticulosis (*n* = 3), endometriosis (*n* = 2), anal fissure (*n* = 1), post‐episiotomy (*n* = 3), obstetrical rectal tear (*n* = 1), anorectal cleansing (*n* = 1). Two patients with OASIS were excluded because they were exclusively treated by a diverting stoma.

Only the 60 patients with treated ARVF due to OASIS were considered in this study. The mean age of these 60 patients was 36.2 (±10.6) years and the mean BMI was 27 kg/m^2^ (±5.5). The OASIS had a grade 3 in six patients and a grade 4 in 19 patients. The grade of OASIS was not reported in 35 women.

Among the 60 patients, 148 surgeries were performed, accounting for a mean of 2.5 (±1.7) surgeries per patient. A diverting stoma procedure was performed in 29 patients (48.3%) of which 26 were closed at the end of the management period (89.7%). The overall management was successful in 55 patients (91.7%) after a mean follow‐up of 12 months (±18) from the date of the last surgery.

### Description of surgery cohort

As is shown in Table 1, 55 procedures were successful (37.2%). Of the 148 procedures performed, 70 were performed in association with a diverting stoma (47.3%).

**TABLE 1:** Characteristics of the procedures according to the successfulness or unsuccessfulness of the procedure

	Unsuccessful procedure *n* = 93 (62.8%)	Successful procedure *n* = 55 (37.2%)	Overall procedures (*n* = 148)	*p*‐value
Diverting stoma	45 (48.4%)	25 (45.4%)	70 (47.3%)	0.73
Surgical procedure				
Endorectal advancement flap	38 (42.2%)	11 (20%)	49 (33.1%)	0.09
Musset procedure	8(8.9%)	15 (27.3%)	23 (15.9%)	
Martius flap	8 (8.9%)	8 (14.5%)	16 (11%)	
Seton ablation	10 (11.1%)	4 (7.3%)	14 (9.7%)	
Insertion of glue	6 (6.7%)	4 (7.3%)	10 (6.9%)	
Ovesco clip	6 (6.7%)	3 (5.4%)	9 (6.2%)	
Vaginal suture or vaginal flap	5 (5.6%)	3 (5.08%)	8 (5.5%)	
Plug	4 (4.4%)	3 (5.4%)	7 (4.8%)	
Gracilis flap	1 (1.1%)	2 (3.6%)	3 (2.1%)	
Other procedures	4 (4.4%)	2 (5.4%)	6 (4.1%)	
Rank of the procedure	2.33 + −1.81	2.38 + −1.71	2.35 + −1.76	0.87

*Note:* The percentages were calculated by using only the data available.

The endorectal advancement flap was the most performed procedure (*n* = 49, 33.1%). It was also the most performed procedure of the failed procedures (42.2%) and was a less frequently performed procedure of the successful procedures (20%; *p* = 0.09). The Musset procedure and the Martius flap were more frequently performed in the successful group (27.3 and 14.5%, respectively) than in the group of failed procedures (8.9 and 8.9%, respectively). The other six overall procedures in Table 1 included two fistulotomies and each one of seton traction, vaginal and clip, pudendal flap and myorraphy of the levator ani.

There was no significant difference in the rate of diverting stoma for the group of successful procedures (45.4%) and that of the group of failed procedures (48.4%; *p* = 0.73; Table 1).

### Description of patient cohort

There was no significant difference between the patients experiencing successful or unsuccessful management with respect to their age, BMI, status as being overweight, history of Crohn's disease or proctological surgery history (Table 2). The mean number of surgical procedures performed and the mean number of surgical procedures performed protected by a diverting stoma were also not significantly different between the two groups of patients.

**TABLE 2:** Characteristics of the patients and of the surgical management, according to the patient

	Unsuccessful overall management *n* = 5 (8.3%)	Successful overall management *n* = 55 (91.7%)	Overall population (*n* = 60)	*p*‐value
Mean age, years	33.6 + −8.7	36.4 + −10.7	36.1 + −10.6	0.58
BMI > 25 kg/m^2^	3 (60%)	26 (57.8%)	29 (58%)	0.92
BMI, kg/m^2^	27.6 + −6.6	26.9 + −5.5	26.9 + −5.5	0.79
Proctologic medical history	1 (20%)	31 (56.4%)	32 (53.3%)	0.12
Medical history of Crohn's disease	0 (0%)	2 (5.9%)	2 (5.3%)	0.62
Parity	1.2 + −0.4	1.6 + −1.6	1.5 + −1.5	0.61
Mean number of surgical procedures performed	3.61 + −2.1	2.40 + −1.7	2.5 + −1.7	0.14
Patients having at least one procedure protected by stoma	3 (60%)	26 (47.3%)	31 (51.7%)	0.59
Mean percentage of surgical procedures performed with a concomitant diverting stoma	0.4 + −0.5	0.4 + −0.4	0.4 + −0.4	0.80
Mean number of surgical procedures performed with a concomitant diverting stoma	2 + −2	1.1 + −1.9	1.2 + −1.9	0.31

### Multivariate analysis of predictive factors of success

In multivariate analysis, the number of recurrences prior to surgical treatment was not associated with a variation in the treatment's success rate (OR = 1.37; 95% CI: 0.75–2.51) (Table 3). Performing a diverting stoma procedure did not improve the efficacy of surgery (OR = 1.46; 95% CI: 0.31–6.91). There was no interaction between the number of recurrences and the performance of a diverting stoma procedure (*p* = 0.34; Table 3); the impact of a diverting stoma did not vary depending on the number of recurrences prior to surgical treatment. However, compared to the advancement rectal flap, the Martius flap procedure increased management efficacy (OR = 4.13; 95% CI: 1.1–15.54), as did the Musset procedure (OR = 5.79; 95% CI: 1.77–18.87).

**TABLE 3:** Multivariate analysis of the predictive factors for successful surgery

	Odds ratio	95% confidence interval	*p*‐value
Diverting stoma	1.46	0.31–6.9	0.64
Procedure (ref: ERAF)			
Ablation of the seton	1.13	0.23–5.53	0.88
Glue	2.19	0.43–11.2	0.35
Martius flap	4.13	1.1–15.54	0.04
Vaginal flap	2.46	0.44–13.71	0.31
Musset procedure	5.89	1.77–18.87	0.004
Other	1.81	0.56–5.78	0.32
Rank of the surgery	1.37	0.75–2.51	0.29
Interaction between the diverting stoma and the rank of the surgery	0.73	0.38–1.39	0.34
BMI > 25 kg/m^2^	0.71	0.31–1.60	0.41

Abbreviation: ERAF, endorectal advancement flap.

## DISCUSSION

Among this group of 60 ARVF patients, the overall management success rate was 91.7%. The women underwent a mean of 2.5 (±1.7) procedures. A diverting stoma procedure was performed in 29 patients (48.3%), of which 26 were closed at the end of the management period (89.7%).

Of the 148 surgical procedures, only 55 were successful (37.2%). The order of the procedures was not significantly associated with the success of the surgery as well as that of the diverting stoma. However, the Martius flap and Musset procedures had better results than the rectal flap. The other procedures were not significantly associated with the success of management.

First, we reported that 18.7% of the overall population of those with ARVF have ARVF due to OASIS. This is similar to that found in the literature, which reports rates of ARVF due to OASIS of between 9% and 34% [6, 8, 9, 14]. The present series has the advantage of exclusively focusing on ARVF and of being the largest series of ARVF due to OASIS. Indeed, to the best of our knowledge, the only other large series that focuses on post‐obstetrical fistula is the series reported by Sjoveian et al. [15], which included 595 patients operated on in eastern Democratic Republic of Congo over a 3‐year period. However, this series aims to assess predictors of surgical outcomes, focusing only on vesicovaginal fistulas without taking into account ARVF.

Second, this study aimed to assess the effect of diverting stoma in improving the success of the surgical procedure. In our series, 48.3% of the patients received a diverting stoma. This is higher than the rate reported in the only study focusing exclusively on ARVF following OASIS, where it is stated that only 25% of patients required a stoma [16]. We are unable to explain why more stoma procedures were performed in our study, unless this can be attributed to the fact that the centres treating the patients who were included in the study were level 3 centres and the population of patients may present greater complexities than the patients at other centres. While the cases of these patients may be more complex, our results did not highlight any improvement linked to a diverting stoma or the point in time at which the procedure is carried out. This provides evidence for arguing that procedures for diverting stoma should not be systematically performed on those patients. The literature shows a different conclusion and in a recent study reporting 79 patients with ARVF, including only 9% ARVF due to OASIS, the diverting stoma improved the results of surgery (OR = 3.2; 95% CI: 1.5–9.6). This difference is probably due to the fact that this series included 43% ARVF due to Crohn's disease and 32% postoperative ARVF for which stoma is likely to be necessary [6]. Indeed, Karp et al. [14] report that nonobstetric fistulas have a near 4‐fold increased risk of recurrence compared to obstetric fistulas. Also, in another series, the authors did not highlight any improvement of the success rate associated with a diverting stoma [7]. In the latter series, the authors reported no ARVF due to Crohn's disease and a low rate of post‐surgical ARVF (28.6%). The authors of this series concluded that a diverting stoma could be necessary for patients in whom the first operation failed. This last statement is not supported by our results because the point at which the stoma was formed did not improve the result of surgery.

However, the stoma should probably not be abandoned because, even if it does not improve the results of surgery, it may improve patients' quality of life by diverting stools and flatus and preventing emissions from the vagina. Only few studies assessed the effect of diverting stoma on the quality of life of women experiencing ARVF. In 2007, Kasparek et al. [17] compared the quality of life of Crohn's disease women with diverted ARVF and undiverted ARVF. They concluded that the quality of life seemed to be similar or potentially superior in diverted ARVF. Such a study has not been performed in OASIS patients but diverting stoma may improve patients' quality of life. Also, Garg et al. [18] reported that the other aim of a diverting stoma is to reduce the risk of septicaemia. These two other aims of diverting stoma have to be discussed with the patient in order to decide the management of the ARVF.

In our series, neither the order in which the surgery was performed nor the recurrence were risk factors for failure of the surgery. This was not supported in the literature, which reports recurrence as a risk factor for failure of surgery [7]. In the latter series, only 28% of the patients had ARVF due to OASIS. This could explain the difference.

Finally, we highlighted that Martius flap (50%) and Musset procedures (65.2%) had better results than the endorectal advancement flap procedure (22.4%). Interestingly, the endorectal advancement flap is the most performed procedure. This is in accordance with the recommendation that such a procedure be performed as a primary intervention [10]. Studniarek et al. [9] recommend carrying out the endorectal flap procedure in cases of low‐lying, simple ARVF as is generally the case in instances of OASIS. However, they do not differentiate between situations in which the sphincters retain their integrity and those in which they are disrupted. In the first situation, the endorectal advancement flap procedure could probably be performed safely, but the patient should be informed of the risk of failure. The Martius flap procedure could probably be performed as a second step, as it offers better results in cases of greater injury. In cases of sphincter disruption, the Musset procedure should probably be performed as a first intervention because it offers better results than the advancement endorectal flap procedure.

However, there are some limitations inherent in our study's retrospective approach. Specifically, there is no specific code for ARVF and some patients could be missing. Additionally, there was no data on perioperative management (antibiotics, laxative treatments, bowel preparation…). These parameters are not consensual and Dawes et al. report in their mini‐review differing perioperative management among the patients undergoing an endorectal advancement flap procedure [4]. Also, some information such as the diameter or the location of the ARFV was missing in our series. However, it has not previously been reported to influence the success rate [7]. Diabetes or smoking were also not collected which could influence the success rate of healing. Also, the examinations performed before surgery were not collected.

Despite these limitations, our study is one of the largest series of ARVF post‐OASIS and provides some strong evidence, particularly in regard to the fact that procedures to create diverting stoma should not be performed at any time in order to improve the rate of healing.

## CONCLUSION

The management of ARVF following OASIS is complicated and leads to a high risk of recurrence. The creation of a diverting stoma is not essential because it does not improve the success rate of surgery, but it could be performed in order to improve patients' quality of life and reduce the symptoms. An endorectal flap could be a good option for initial treatment of ARVF, but in cases of failure the Martius flap or Musset procedure should probably be performed.

## AUTHOR CONTRIBUTIONS

*Conception*: Aurélien Venara, Jean‐Francois Hamel; Planning: Aurélien Venara, Jean‐Francois Hamel, Bertrand Trilling; *Execution*: Aurélien Venara, Bertrand Trilling, Marie Ngoma, Charlène Brochard, Emilie Duchalais, Laurent Siproudhis, Jean‐Luc Faucheron, Vincent de Parades, Arnaud Alves, Eddy Cotte, Mehdi Ouaissi, Valérie Bridoux, Lisa Corbière, Jeanne Heraud, Pablo Ortega‐Deballon, Fawaz Abo‐Alhassan, Jean‐Francois Hamel; Analysis: Aurélien Venara, Jean‐Francois Hamel; *Writing*: Aurélien Venara, JFH; *Critical revision of manuscript for important intellectual content*: Aurélien Venara, Bertrand Trilling, Marie Ngoma, Charlène Brochard, Emilie Duchalais, Laurent Siproudhis, Jean‐Luc Faucheron, Vincent de Parades, Arnaud Alves, Eddy Cotte, Mehdi Ouaissi, Valérie Bridoux, Lisa Corbière, Jeanne Heraud, Pablo Ortega‐Deballon, Fawaz Abo‐Alhassan, Jean‐Francois Hamel; *Final approval of the version to be published*: Aurélien Venara, Bertrand Trilling, Marie Ngoma, Charlène Brochard, Emilie Duchalais, Laurent Siproudhis, Jean‐Luc Faucheron, Vincent de Parades, Arnaud Alves, Eddy Cotte, Mehdi Ouaissi, Valérie Bridoux, Lisa Corbière, Jeanne Heraud, Pablo Ortega‐Deballon, Fawaz Abo‐Alhassan, Jean‐Francois Hamel.

## CONFLICT OF INTEREST

Venara declares conflicts of interest with Takeda, Coloplast, ThermoFisher, Biom'up, Sanofi‐Aventis (consulting and lecture). The other authors do not declare any conflict of interest.

## ETHICAL STATEMENT

The study received the approval of our local ethical committee (2021/034) and the database was declared tot he comission national informatique et libertés (ar21‐0014v0).

## INFORMED CONSENT

Patients were orally informed that their data could be used for retrospective studies. They were asked to inform the staff if they disagree with the use of their data.

## Data Availability

Research data are not shared.
